# Supplementary material for: Hypoxia Exacerbates Inflammatory Signaling in Human Coronavirus OC43-Infected Lung Epithelial Cells
Source: Biomolecules. 2025 Aug 8;15(8):1144. doi: 10.3390/biom15081144 (PMC12384405; doi:10.3390/biom15081144)
Supplement: Supplementary file 1 [file biomolecules-15-01144-s001.zip › biomolecules-3736977-Supplementary Figure S1.pdf]

Supplementary Figure 1

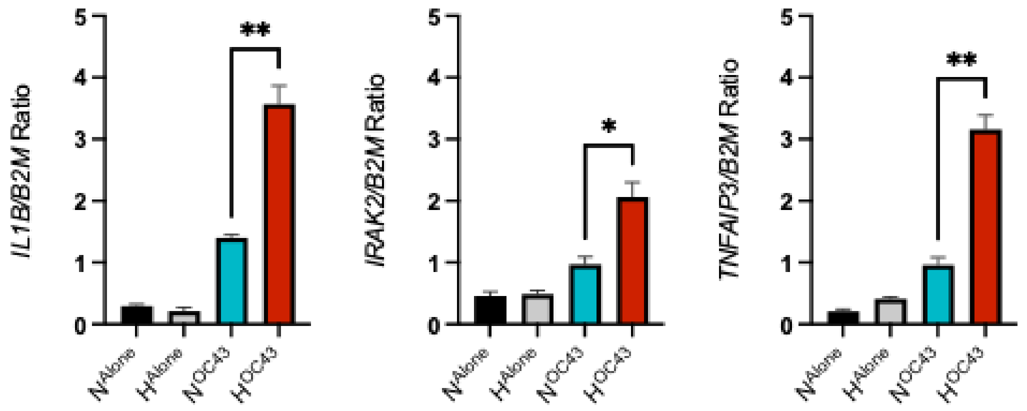

Figure S1 – Analysis of synergistic gene expression in MRC-5 cells by HCoV-OC43 infection in hypoxia. MRC-5 cells were infected with HCoV-OC43 at an MOI of 5 for 24 h in hypoxia or normoxia. The graphs show the gene expression of (**A**) *IL1B*, (**B**) *IRAK2* and (**C**) *TNFAIP3* gene expression at 24 hpi. The *IL1B/B2M*, *IRAK2/B2M* and *TNFAIP3/B2M* ratios were calculated by taking the starting concentrations of the mRNAs of the genes of interest, obtained from the qRT-PCR run of the samples, and dividing them by the starting concentration of B2M mRNA. An unpaired T-test was used to assess significance (Graphpad Prism v10) * $p = < 0.05$, ** $p = < 0.01$. Error bars display the SEM (n = 3 triplicate).
