# Supplementary material for: Hypoxia Exacerbates Inflammatory Signaling in Human Coronavirus OC43-Infected Lung Epithelial Cells
Source: Biomolecules. 2025 Aug 8;15(8):1144. doi: 10.3390/biom15081144 (PMC12384405; doi:10.3390/biom15081144)
Supplement: Supplementary file 1 [file biomolecules-15-01144-s001.zip › biomolecules-3736977-Supplementary Figure S2.pdf]

Supplementary Figure 2

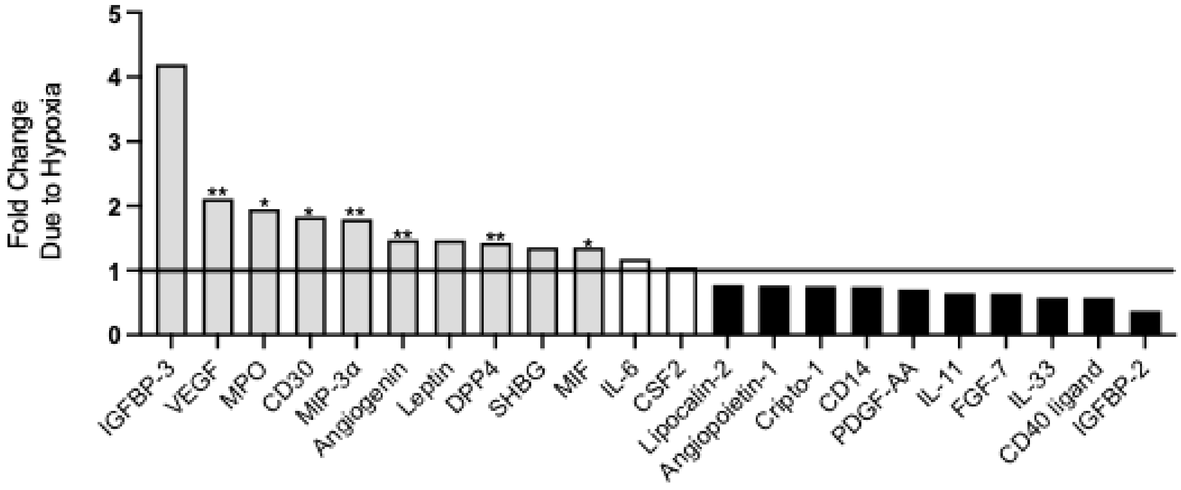

Figure S2 – Analysis of IRES sequences in mRNAs coding for proteins that are up-regulated or down-regulated due to OC43 infection in hypoxia. The light grey bars represent the top 10 proteins that were upregulated in the H^{OC43} condition compared to N^{OC43}. The black bars represent the top 10 proteins that were in the downregulated in the H^{OC43} condition compared to N^{OC43}. The white bars represent two inflammatory cytokines of interest that have been associated with cytokine storms in patients with severe COVID-19. These two cytokines also exhibited significant synergistic upregulation in our RNA sequencing data. The Human IRES Atlas is a tool that can predict IRES sequences in the 5′ UTR and assess how likely an identified IRES is to impact the translation of a protein (Yang et al., 2021). This prediction is represented in the RNA-protein interaction (RPI) score, a score that uses twelve different RNA structure prediction tools and processes the data to give an RPI significance value. * Represents identification of an IRES using the Human IRES Atlas. ** Represents identification of an IRES using the Human IRES Atlas that also has an RPI value less than $1x10^{-6}$, indicating a higher probability of the IRES sequence being functional.

Yang, T.H., Wang, C.Y., Tsai, H.C., Liu, C.T., 2021. Human IRES Atlas: an integrative platform for studying IRES-driven translational regulation in humans. Database (Oxford) 2021.
